# Editorial to “Management of refractory intramural LV summit ventricular arrhythmia: Acute success using bipolar radiofrequency catheter ablation with recurrence”

**DOI:** 10.1002/joa3.13014

**Published:** 2024-02-28

**Authors:** Piotr Futyma, Stefano Bordignon

**Affiliations:** ^1^ St. Joseph's Heart Rhythm Center Rzeszów Poland; ^2^ Medical College University of Rzeszów Rzeszów Poland; ^3^ Cardioangiologisches Centrum Bethanien (CCB), Kardiologie, Medizinische Klinik III, Agaplesion Markus Krankenhaus, Akademisches Lehrkrankenhaus Der Goethe‐Universität Frankfurt Am Main Frankfurt am Main Germany

**Keywords:** advanced ablation techniques, bipolar ablation, left ventricular summit, ventricular tachycardia

The complex architecture of the left ventricular summit (LVS) makes the radiofrequency catheter ablation (RFCA) of ventricular arrhythmias (VA) in that area sometimes difficult.^1^ Quite frequently, a multisite ablation from the LVS adjacent sites is necessary to achieve complete elimination of the LVS VA.^2^ Nevertheless, even such sequential multisite ablation may fail to durably eliminate the clinical arrhythmia. The presence of fat, fibrotic components of cardiac valves, and major coronary vessels frequently prevents thermal energy from full transmural penetration of the targeted LVS sites. In such circumstances, some bailout strategies are implemented into clinical practice to robust ablation outcomes.^3^

One of these strategies involves a bipolar radiofrequency catheter ablation (Bi‐RFCA), when there are two separate ablation catheters connected into the radiofrequency circuit, with one of these catheters connected instead of a dispersive patch electrode. Bi‐RFCA, initially implemented for treatment of posteroseptal accessory pathways and septal VAs, can provide such opportunity in the setting of refractory LVS arrhythmias. The assemblage of sites around LVS area creates a variety of possibilities for two ablation catheters, which can be adjusted in different configurations and orientations. Some of these configurations may include positioning of one of these catheters in the coronary veins.^4^

In the issue of *Journal of Arrhythmia* Sudo et al.^1^ presented an intriguing case report on the application of Bi‐RFCA in the treatment of challenging arrhythmia from the LVS region. The presented patient underwent a repeat ablation procedure using Bi‐RFCA after previously unsuccessful attempts with classic approach for non‐sustained ventricular tachycardia (nsVT) runs and overall significant VA burden. Interestingly, mapping features demonstrated no impressively early activation patterns. Only coronary venous system mapping, performed with a microcatheter, showed some early signals in the lateral vein. Next, the bipolar ablation at moderate power settings between 20 and 30 W was performed between the lateral vein ostium and the opposite endocardial sites. This led to acute elimination of clinical VAs. This acute effect did not come without issues: Due to frequent temperature increase in the catheter located in the lateral vein, the authors were forced to perform multiple applications, which end up with total bipolar ablation time exceeding 17 min. During follow‐up, no nsVT runs were observed; however, the total PVC burden still remained substantial.

The report prompts the discussion regarding several aspects of ablative therapy for LVS arrhythmias. First, it highlights the need for access to advanced ablation strategies, which are sometimes necessary. Second, non‐inducibility of clinical arrhythmia may not always reflect the mid‐ and long‐term outcomes of ablation in the LVS area. Third, despite the presence of substantial VA burden during follow‐up, the clinical outcome was augmented by effective elimination of nsVT episodes.

Bipolar ablation in this instance was not characterized by a clear‐cut binary outcome. The actual clinical benefit was indeed observed in terms of symptomatic relief and the absence of nsVT. However, follow‐up observation critically examines the residual arrhythmia burden, challenging the notion of complete success associated with bipolar RFCA in this particular case. This should prompt a reevaluation of acute outcomes associated with this emerging treatment strategy. Perhaps, intraprocedural measures other than acute VA suppression, such as intramural non‐excitability of targeted area and impedance drop, should help in further improvement in outcome determination in such challenging scenarios?

Radiofrequency ablation is not more the only energy source to perform catheter ablation, and the advent of new technologies using pulsed field ablation (PFA) may impact the results of catheter ablation of ventricular arrhythmias in the future. However, the observation that PFA can induce coronary spasms limits its use in a region such as LVS that is per definition surrounded by coronaries.

It should be acknowledged that the optimal management strategy in cases of more lateral LVS is sought. The size and the tip of ablation catheter positioned in the venous system can play a crucial role in effective energy transfer towards LVS, and the use of large‐tip catheters can help to optimize the impedance of bipolar ablation system.^4^ Also, such ECG features of clinical VA as Q‐wave ratio in leads aVL/aVR >1.85, R/S wave ratio in lead V1 >2, and lack of initial q wave in lead V1 can be suggestive for a relatively rare but possible situation of LVS arrhythmias ablatable from the pericardial space.^5^ These aspects can further improve outcomes of challenging LVS arrhythmia therapies.

The authors should be congratulated for making their efforts in tackling the refractory arrhythmia in the presented patient. Further improvement in understanding of VA clinical features originating from the lateral aspect of the LVS will help to optimize future treatment strategies.

## CONFLICT OF INTEREST STATEMENT

Dr. Futyma reports patents related with bipolar ablation and high‐voltage ablation and that he has equity in CorSystem. Dr. Bordignon reports speaking honoraria from Biosense Webster, Boston Scientific and Medtronic.
